# A multivariable model of ultrasound and clinicopathological features for predicting axillary nodal burden of breast cancer: potential to prevent unnecessary axillary lymph node dissection

**DOI:** 10.1186/s12885-023-11751-z

**Published:** 2023-12-21

**Authors:** Lei Yang, Yifan Gu, Bing Wang, Ming Sun, Lei Zhang, Lei Shi, Yanfei Wang, Zheng Zhang, Yifei Yin

**Affiliations:** 1 Department of Medical Ultrasound, Affiliated Hospital of Nantong University, Medical School of Nantong University, Nantong, 226006 People’s Republic of China; 2 Department of Medical Ultrasound, Affiliated Hospital of Jiangsu University, Zhenjiang, 212000 People’s Republic of China

**Keywords:** Breast cancer, Nodal burden, Clinicopathologic characteristics, Ultrasound features

## Abstract

**Background:**

To develop a clinical model for predicting high axillary nodal burden in patients with early breast cancer by integrating ultrasound (US) and clinicopathological features.

**Methods and materials:**

Patients with breast cancer who underwent preoperative US examination and breast surgery at the Affiliated Hospital of Nantong University (centre 1, *n* = 250) and at the Affiliated Hospital of Jiangsu University (centre 2, *n* = 97) between January 2012 and December 2016 and between January 2020 and March 2022, respectively, were deemed eligible for this study (*n* = 347). According to the number of lymph node (LN) metastasis based on pathology, patients were divided into two groups: limited nodal burden (0–2 metastatic LNs) and heavy nodal burden (≥ 3 metastatic LNs). In addition, US features combined with clinicopathological variables were compared between these two groups. Univariate and multivariate logistic regression analysis were conducted to identify the most valuable variables for predicting ≥ 3 LNs in breast cancer. A nomogram was then developed based on these independent factors.

**Results:**

Univariate logistic regression analysis revealed that the cortical thickness (*p* < 0.001), longitudinal to transverse ratio (*p* = 0.001), absence of hilum (*p* < 0.001), T stage (*p* = 0.002) and Ki-67 (*p* = 0.039) were significantly associated with heavy nodal burden. In the multivariate logistic regression analysis, cortical thickness (*p* = 0.001), absence of hilum (*p* = 0.042) and T stage (*p* = 0.012) were considered independent predictors of high-burden node. The area under curve (AUC) of the nomogram was 0.749.

**Conclusion:**

Our model based on US variables and clinicopathological characteristics demonstrates that can help select patients with ≥ 3 LNs, which can in turn be helpful to predict high axillary nodal burden in early breast cancer patients and prevent unnecessary axillary lymph node dissection.

## Background

Axillary lymph node (ALN) status is a critical prognostic factor in the therapeutic plan of breast cancer patients because it can determine the extent of surgery and assess the necessity of chemotherapy or radiotherapy [1–4]. Axillary lymph node dissection (ALND) is used to assess ALN status, provide accurate staging of axillary lymph nodes and eliminate potential metastatic lymph nodes, and it is the standard surgical method for patients associated with greater lymph node burden [3–6]. However, ALND can cause severe complications, such as shoulder dyskinesia and arm lymphedema, which can have a negative impact on quality of life [7–12]. Hence, avoiding excessive ALND becomes a pressing issue. The results of the ACOSOG Z0011 trial showed no statistically significant differences between the ALND and no-ALND groups in terms of local recurrence rate and 10-year overall survival rate in patients with < 3 axillary lymph node metastases [13]. Patients with ≥ 3 axillary lymph node metastases (high axillary nodal burden) are more likely to have local recurrence, and are thus considered suitable for neoadjuvant chemotherapy or ALND [14–17]. Therefore, preoperative prediction of lymph node metastasis and identification of patients with high axillary nodal burden are crucial to determine the appropriate therapeutic management.

Preoperative imaging, including ultrasound (US), mammography, computed Tomography, magnetic resonance imaging, positron emission tomography etc., has become increasingly important and more widely used in assessing ALN metastasis in patients with breast cancer [18–21]. Compared with other imaging modalities, ultrasound is more cost-effective, non-invasive and reproducible [14, 15, 22]. However, Hieken et al*.* showed a false positive rate of 79.8% for suspicious axillary ultrasound results according to pathological examination [23]. Therefore, it is insufficient to assess ALN burden by axillary ultrasound alone. Fortunately, the US characteristics of primary breast lesion are reported to be obviously associated with high ALN metastasis [24, 25]. In addition, certain clinicopathological features of patients with breast cancer are related to ALN metastasis [26–28]. The purpose of this study was to integrate the ultrasonic features of lymph nodes and primary lesions with clinicopathological characteristics to identify independent predictors and develop a model to predict high-burden lymph node (≥ 3) in patients with breast cancer and prevent unnecessary ALND.

## Materials and methods

This study design followed the international regulations according to the Declaration of Helsinki. Our research was approved by the Ethical Committee of the Affiliated Hospital of Nantong University (2022-K108-01) and Affiliated Hospital of Jiangsu University (KY2021K1213), and written informed consent was obtained from participants.

### Patient enrolment

**Fig. 1:**
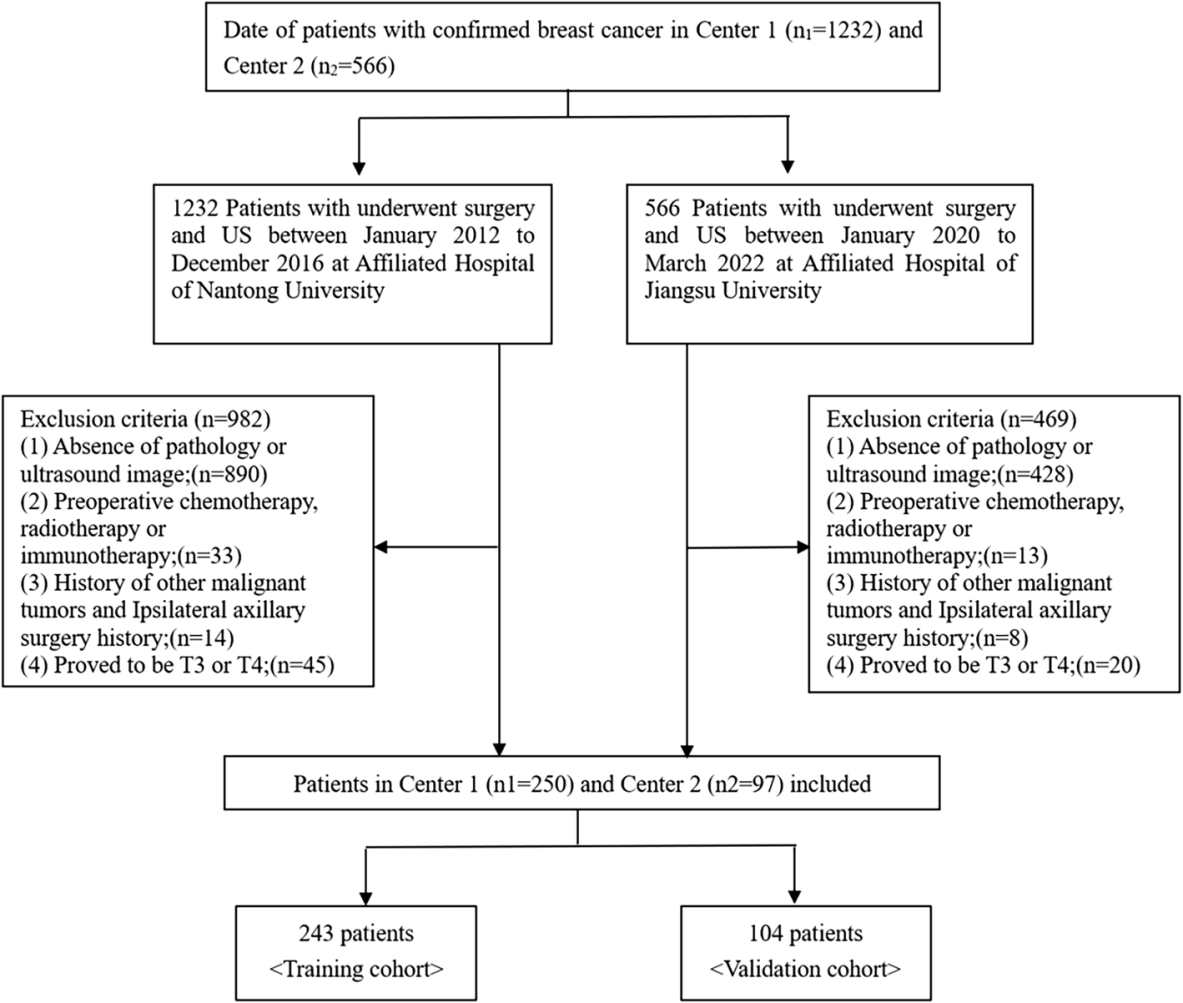
Flowchart of the study population

Patients who underwent breast surgery and US examination at the Affiliated Hospital of Nantong University (centre 1, *n* = 1232) and at the Affiliated Hospital of Jiangsu University (centre 2, *n* = 566) between January 2012 and December 2016 and between January 2020 and March 2022, respectively, were deemed eligible for this study (*n* = 1798). Patients were included if they: [1] had clinical N1 or N0; [2] had a preoperative breast US and axillary US performed within two weeks of surgery, which recorded US characteristics of the primary breast tumour and axillary lymph nodes; [3] had breast surgery and axillary lymph node dissection; [4] had pathology which documented the number of axillary lymph node metastasis; [5] and had a pathology tumour size that was T1 or T2. Exclusion criteria were as follows: [1] patients with a history of other malignant tumours and ipsilateral axillary surgery history; [2] patients who underwent preoperative chemotherapy, radiotherapy or immunotherapy and [3] absence of clinicopathological or US information. Finally, 347 patients were included in this study (Fig. 1).

### US analysis

All patients included in this study underwent a preoperative breast and axillary US examination within two weeks of surgery using the GE LOGIQ E9 with a linear array transducer (12–15 MHz). Patients were positioned flat with bilateral arms raised to fully expose the bilateral breasts and axilla. US parameters, such as gain, depth, focal length, etc., were adjusted to enable a clear display of the primary lesion. The primary lesion or lymph node used for the assessment was situated in the central part of the ultrasound screening. Subsequently, we scanned the lesion from multiple angles and acquired images of the primary lesion and lymph nodes. The images were then stored for further analysis.

US features of the primary tumour and ALNs were observed independently by two radiologists who were blinded to any information that could interfere or bias with their task. Potential disagreements or differences were arbitrated by a third experienced radiologist to reach a consensus. The US features of the primary tumour were analysed, including quadrants (upper outer, upper inner, lower outer and lower inner quadrants), margins (circumscribed or non-circumscribed), orientation (parallel or non-parallel), shape (regular or irregular), attenuation (weak or not) and calcification (with or without). Furthermore, axillary US measured the longitudinal to transverse ratio (< 2 or ≥ 2) and cortical thickness (< 3 mm or ≥ 3 mm), and determined the absence of hilum (yes or not).

### Clinicopathological analysis

Surgical-histopathologic data included the number of metastatic lymph nodes (< 3 or ≥ 3), T stage (≤ 2 cm or 2–5 cm), histological grade (I, II or III) and the expression of estrogen receptor (ER), progesterone receptor (PR), CerbB-2, P53 and Ki-67 from histopathology reports. ER and PR positivity were defined as the expression of greater than 1% [29], while Ki67 positivity was defined as the expression of greater than 14% [30]. CerbB-2 receptor of 3 + in HE or 2 + in gene amplification was defined as human epidermal growth factor receptor 2 (HER-2) positive [29, 31].

### Statistical analysis

Statistical analysis was conducted using the SPSS software (ver. 24.0; SPSS Inc., Chicago, IL, USA), the MedCalc software (ver.19.07) the R software (ver 4.0.1). The enrolled patients were randomly divided into the training group and the verification group according to the ratio of 7:3. X2 tests or Fisher’s exact test were performed between the training and the validation groups. Univariate logistic regression analysis was used to identify factors that could significantly affect the training group. Multivariate logistic regression analysis was applied to determine independent predictors of the number of axillary lymph node metastasis and incorporate them into the model. The area under the area under curve (AUC), accuracy (ACC), sensitivity, specificity, positive predictive value (PPV) and negative predictive value (NPV) was used to assess model discrimination. Delong test was conducted to compare different diagnostic models across the training and validation cohort for nomogram, cortical thickness, lymphatic gate, and T stage by MedCale. Finally, a calibration diagram was drawn to evaluate the ability of calibration and the fit of the model was assessed by the Hosmer–Lemeshow goodness-of-fit test.

## Results

### Baseline characteristics

Table 1 shows the characteristics of the research population. Patients were divided into the training group (*n* = 243) and the verification group (*n* = 104) with a ratio of 7:3. Fifty-six (23.0%) and 30 (28.8%) patients had ≥ 3 lymph node metastases of primary breast cancer in the training and validation cohorts, respectively. There were no significant differences in the US characteristics and clinicopathological parameters between the two groups. The mean ages of the training and validation groups were 55.84 ± 10.74 and 55.54 ± 11.66, respectively.

**Table 1:** Characteristics of the training and validation cohorts

Characteristic	Training Cohort (*n* = 243)	Validation Cohort (*n* = 104)	*P*-value
Age	55.84 ± 10.74	55.54 ± 11.66	0.822
Cortical thickness			0.670
< 3	149(61.3%)	67(64.4%)	
≥ 3	94(38.7%)	37(35.6%)	
Longitudinal to transverse ratio			0.969
≥ 2	145 (59.7%)	63(60.6%)	
< 2	98 (40.3%)	41(39.4%)	
Absence of hilum			0.913
no	191 (78.6%)	83(79.8%)	
yes	52 (21.4%)	21(20.2%)	
Location			0.867
UIQ	39 (16.0%)	15 (14.4%)	
LIQ	16 (6.6%)	7 (6.7%)	
LOQ	47 (19.3%)	17(16.3%)	
UOQ	141 (58.0%)	65 (62.5%)	
Orientation			0.751
Parallel	174 (71.6%)	72 (69.2%)	
Non-parallel	69(28.4%)	32 (30.8%)	
Shape			1
Regular	27 (11.1%)	12 (11.5%)	
Irregular	216 (88.9%)	92 (88.5%)	
Margin			0.316
Circumscribed	41 (16.9%)	23 (22.1%)	
Non-circumscribed	202 (83.1%)	81 (77.9%)	
Calcification			0.264
No	104 (42.8%)	52 (50.0%)	
Yes	139 (57.2%)	52 (50.0%)	
Attenuation			0.830
No	159 (65.4%)	70 (67.3%)	
Yes	84 (34.6%)	34 (32.7%)	
ER			0.800
Negative	89 (36.6%)	34 (32.7%)	
1 +	31(12.8%)	17(16.3%)	
2 +	31(12.8%)	13(12.5%)	
3 +	92(37.9%)	40 (38.5%)	
PR			0.642
Negative	109(44.9%)	43 (41.3%)	
1 +	40(16.5%)	15(14.4%)	
2 +	38(15.6%)	22(21.2%)	
3 +	56(23.0%)	24 (23.1%)	
Her-2			0.879
Negative	195 (80.2%)	82 (78.8%)	
Positive	48 (19.8%)	22 (21.2%)	
P53			0.485
Negative	109 (44.9%)	42 (40.4%)	
Positive	113 (46.5%)	49 (47.1%)	
Unknow	21(8.6%)	13(12.5%)	
Ki-67			0.217
< 14	34 (14.0%)	19 (18.3%)	
≥ 14	204 (84.0%)	85 (81.7%)	
Unknow	5(2.1%)	0(0.0%)	
Tumor size			0.716
T1	119 (49.0%)	48 (46.2%)	
T2	124 (51.0%)	56 (53.8%)	
Histological grade			0.535
I	18 (7.4%)	6 (5.8%)	
II	117 (48.1%)	57 (54.8%)	
III	104 (42.8%)	40 (38.4%)	
Unknow	4(1.6%)	1(1.0%)	

*LN* Lymph node, *UOQ* Upper outer quadrant, *UIQ* Upper inner quadrant, *LOQ* Lower outer quadrant, *LIQ* Lower inner quadrant, *ER* Estrogen receptor, *PR* Progesterone receptor, *HER2* Human epidermal growth factor receptor

### Univariate and multivariate analyses

In the univariate analysis, variables that were significantly associated with ≥ 3 lymph node metastases included cortical thickness (*p* < 0.001), longitudinal to transverse ratio (*p* = 0.001), absence of hilum (*p* < 0.001), T stage (*p* = 0.002) and Ki-67 (*p* = 0.039) (Table 2 and 3). The remaining factors were not found to be significant for the identification of high-burden lymph nodes (all *p* > 0.05). In the multivariate logistic regression analysis, cortical thickness (*p* = 0.001), absence of hilum (*p* = 0.042) and T stage (*p* = 0.012) are shown in Table 4. Ki-67 and longitudinal to transverse ratio were not independent predictors. Cortical thickness, absence of hilum and T stage were considered as independent predictors of HBN, and these parameters were then incorporated into the predictive model to create a nomogram (*p* < 0.05).

**Table 2:** Univariable analysis ultrasound features of lymph nodes in the training cohort

Variable	0 or 1, 2 metastatic LNs (*n* = 187)	≥ 3 metastatic LNs (*n* = 56)	Odds Ratio	95% CI	*P*-value	
Cortical thickness						
< 3	131(70.1%)	18(32.1%)	Reference			
≥ 3	56(29.9%)	38(67.9%)	4.938	2.598–9.386	< 0.000*	
Longitudinal to transverse ratio						
≥ 2	123(65.8%)	22(39.3%)	2.970	1.605–5.497	0.001*	
< 2	64(34.2%)	34(60.7%)	Reference			
Absence of hilum						
No	159(85.0%)	32(57.1%)	Reference			
Yes	28(15.0%)	24(42.9%)	4.259	2.192–8.277	< 0.001*	

*P values less than 0.05

**Table 3:** Univariable analysis of ultrasound and clinicopathological features of primary tumor in the training cohort

Variable	0 or 1, 2 metastatic LNs (*n* = 187)	≥ 3 metastatic LNs (*n* = 56)	Odds Ratio	95% CI	*P*-value	
Age						
< 60	121(64.7%)	34(60.7%)	Reference			
≥ 60	66(35.3%)	22(39.3%)	1.186	0.642–2.193	0.586	
Location						
UIQ	31(16.6%)	8(14.3%)	Reference			
LIQ	13(7.0%)	3(5.4%)	0.812	0.341–1.934	0.638	
LOQ	36(19.3%)	11(19.6%)	0.726	0.195–2.701	0.633	
UOQ	107(57.2%)	34(60.7%)	0.962	0.442–2.093	0.921	
Orientation						
Parallel	137(73.3%)	37(66.1%)	Reference			
Non-parallel	50(26.7%)	19(33.9%)	1.407	0.741–2.671	0.296	
Shape						
Regular	19(10.2%)	8(14.3%)	Reference			
Irregular	168(89.8%)	48(85.7%)	0.679	0.280–1.646	0.391	
Margin						
Circumscribed	35(18.7%)	6(10.7%)	Reference			
Non-circumscribed	152(81.3%)	50(89.3%)	1.919	0.762–4.830	0.166	
Calcification						
No	83(44.4%)	21(37.5%)	Reference			
Yes	104(55.6%)	35(62.5%)	1.330	0.720–2.456	0.362	
Attenuation						
No	128(68.4%)	31(55.4%)	Reference			
Yes	59(31.6%)	25(44.6%)	1.750	0.950–3.222	0.073	
ER						
Negative	69(36.9%)	20(35.7%)	Reference			
1 +	23(12.3%)	8(14.3%)	0.870	0.438–1.727	0.690	
2 +	26(13.9%)	5(8.9%)	1.043	0.411–2.652	0.929	
3 +	69(36.9%)	23(41.1%)	0.577	0.198–1.677	0.312	
PR						
Negative	81(43.3%)	28(50.0%)	Reference			
1 +	30(16.0%)	10(17.9%)	1.267	0.587–2.736	0.546	
2 +	32(17.1%)	6(10.7%)	1.222	0.468–3.189	0.682	
3 +	44(23.5%)	12(21.4%)	0.688	0.233–2.026	0.497	
Her-2						
Negative	150(80.2%)	45(80.4%)	Reference			
Positive	37(19.8%)	11(19.6%)	0.991	0.468–2.100	0.981	
P53						
Negative	81(43.3%)	28(50.0%)	Reference			
Positive	87(46.5%)	26(46.4%)	0.352	0.077–1.613	0.179	
Unknow	19(10.2%)	2(3.6%)	1.157	0.626–2.137	0.642	
Ki-67						
< 14	31(16.6%)	3(5.4%)	Reference			
≥ 14	151(80.7%)	53(94.6%)	0.276	0.081–0.939	0.039*	
Unknow	5(2.7%)	0(0.0%)				
Tumor size						
T1	102(54.5%)	17(30.4%)	Reference			
T2	85(45.5%)	39(69.6%)	2.753	1.454–5.212	0.002*	
Histological grade						
I	16(8.6%)	2(3.6%)	Reference			
II	84(44.9%)	33(58.9%)	3.143	0.685–14.429	0.141	
III	83(44.4%)	21(37.5%)	2.024	0.431–9.498	0.371	
Unknow	4(2.1%)	0(0.0%)				

*LN* Lymph node, *UOQ* Upper outer quadrant, *UIQ* Upper inner quadrant, *LOQ* Lower outer quadrant, *LIQ* Lower inner quadrant, *ER* Estrogen receptor, *PR* Progesterone receptor, *HER2* Human epidermal growth factor receptor *P values less than 0.05

**Table 4:** Comparison of the multivariable in the training cohort

Variable	Odds Ratio	95% CI	*P*-value
Ki-67	0.496	0.135–1.818	0.290
Longitudinal to transverse ratio	1.242	0.581–2.658	0.576
Cortical thickness	3.300	1.598–6.817	0.001*
Absence of Hilum	2.207	1.031–4.727	0.042*
Tumor size	2.389	1.214–4.698	0.012*

*P values less than 0.05

### Development of the nomogram

**Fig. 2:**
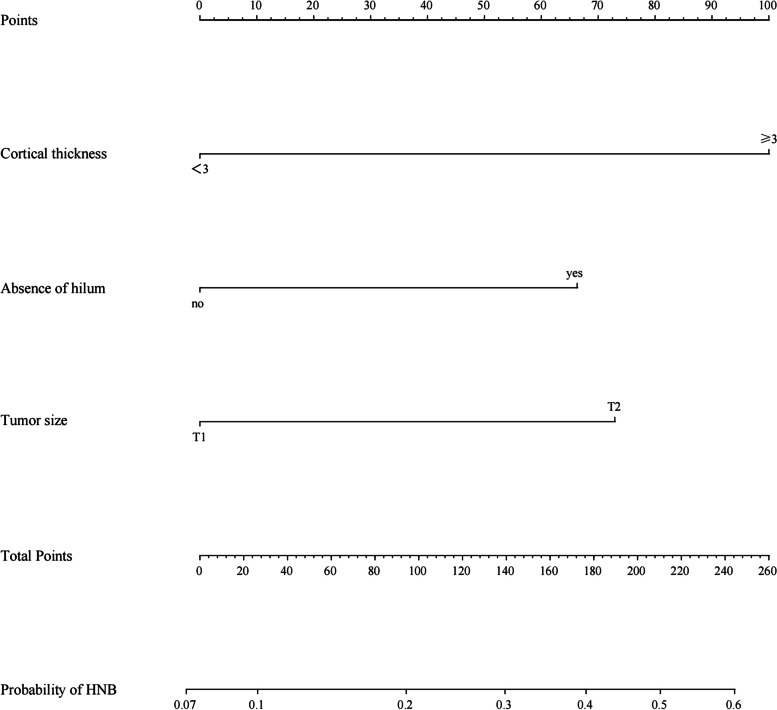
Nomogram for predicting axillary lymph node ≥ 3 metastasis

**Fig. 3:**
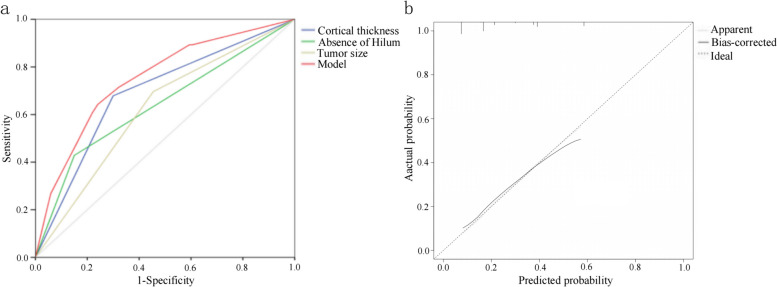
Development and validation of a nomogram to predict axillary lymph node ≥ 3 metastasis. **a** Receiver operating characteristic curves of the model. **b** Calibration plot of the model. In the calibration plot, the dotted line at a 45° angle represents perfect calibration

**Fig. 4:**
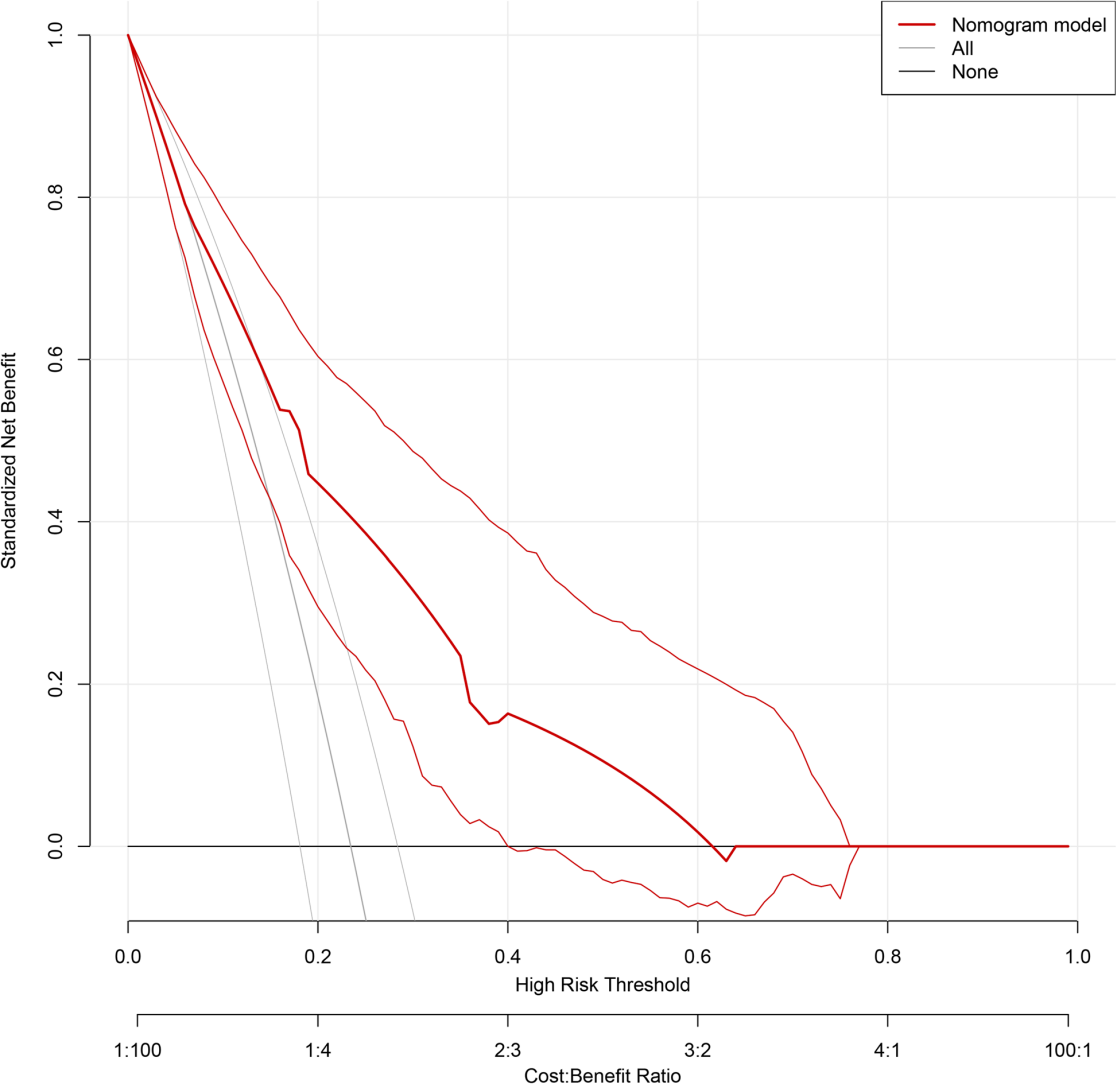
Decision curve analysis for the nomogram model in the training set

Based on the results of the multivariate logistic regression analysis, cortical thickness, absence of hilumand T stage were incorporated to create a nomogram **(**Fig. 2). The nomogram had an AUC of 0.749 (95% CI: 0.676–0.823), sensitivity of 71.4%, specificity of 75.9%, PPV of 67.7% and NPV of 81.1% (Table 5). The AUCs of cortical thickness, lymphatic gate and T stage were 0.690 (95% CI: 0.609–0.770), 0.639 (95% CI: 0.551–0.728) and 0.621 (95% CI: 0.539–0.703), respectively (Fig. 3). The AUC of model was greater than the AUCs of cortical thickness (*p* = 0.003), lymphatic gate (*p* < 0.001) and T stage (*p* < 0.001) (Table 5). The C-index of this model was 0.749 (95% CI: 0.677–0.820). The Hosmer–Lemeshow-Goodness-of-Fit test had a *p*-value of 0.995 and the calibration plot is shown in Fig. 3. Through bootstrap validation, the C-index of the nomogram was considered 0.68. The decision curve analysis (DCA) showed good net benefits in the training set in Fig. 4.

**Table 5:** The ROC analysis of cortical thickness, hilum, T stage and the model in the training cohort

Variable	AUC	ACC(%)	SN (%)	SP (%)	PPV (%)	NPV (%)	*P*-value	95%CI	
								**Lower**	**Upper**
Cortical_thickness	0.690	69.5	67.9	70.1	50.4	87.9	0.003	0.609	0.770
Hilum	0.639	75.3	42.9	85.0	56.1	83.2	< 0.001	0.551	0.728
T stage	0.621	58.0	69.6	54.5	41.5	85.7	< 0.001	0.539	0.703
Model	0.749	78.6	71.4	75.9	67.7	81.1		0.676	0.823

*AUC* Area under curve, *ACC* Accuracy, *SN* Sensitivity, *SP* Specificity, *PPV* Positive Predictive Value, *NPV* Negative Predictive Value, P-value: DeLong test of AUC, *CI* Confidence interval

### Validation of the nomogram

**Fig. 5:**
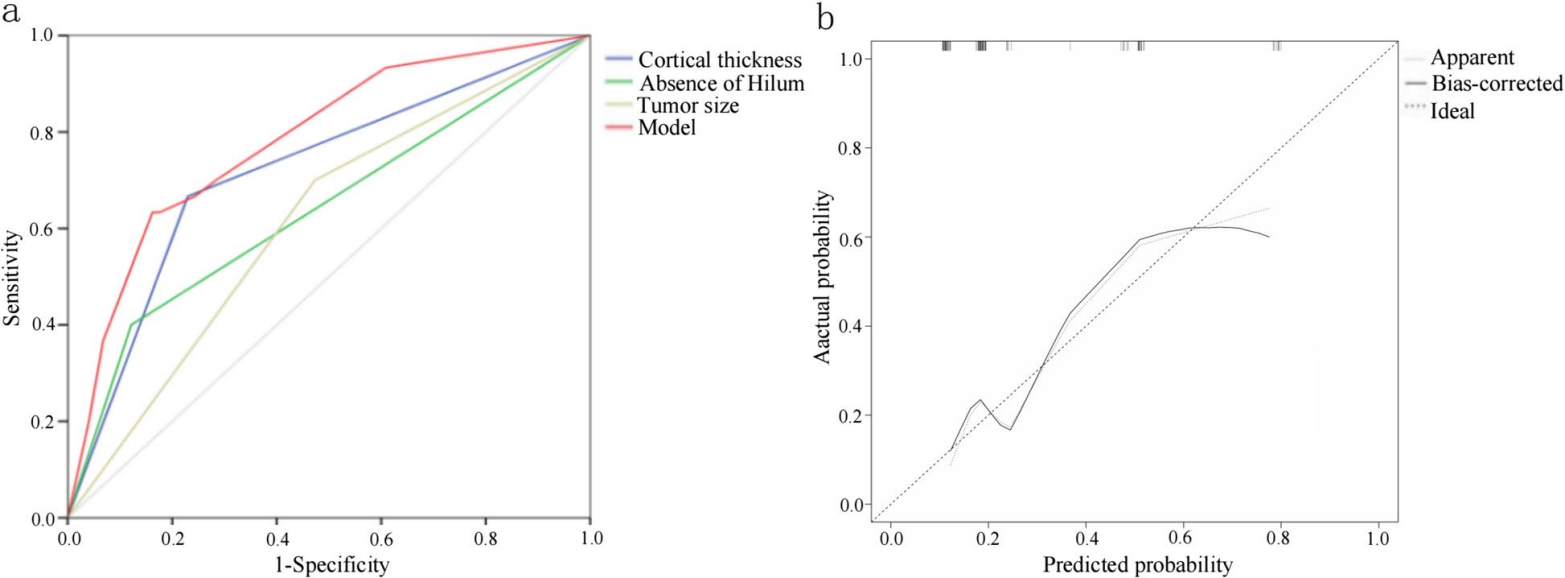
Validation of the nomogram to predict axillary lymph node ≥ 3 metastasis. **a** Receiver operating characteristic curves of the model in validation cohort. **b** Calibration plot of the model in validation cohort. In the calibration plot, the dotted line at a 45° angle represents perfect calibration

**Fig. 6:**
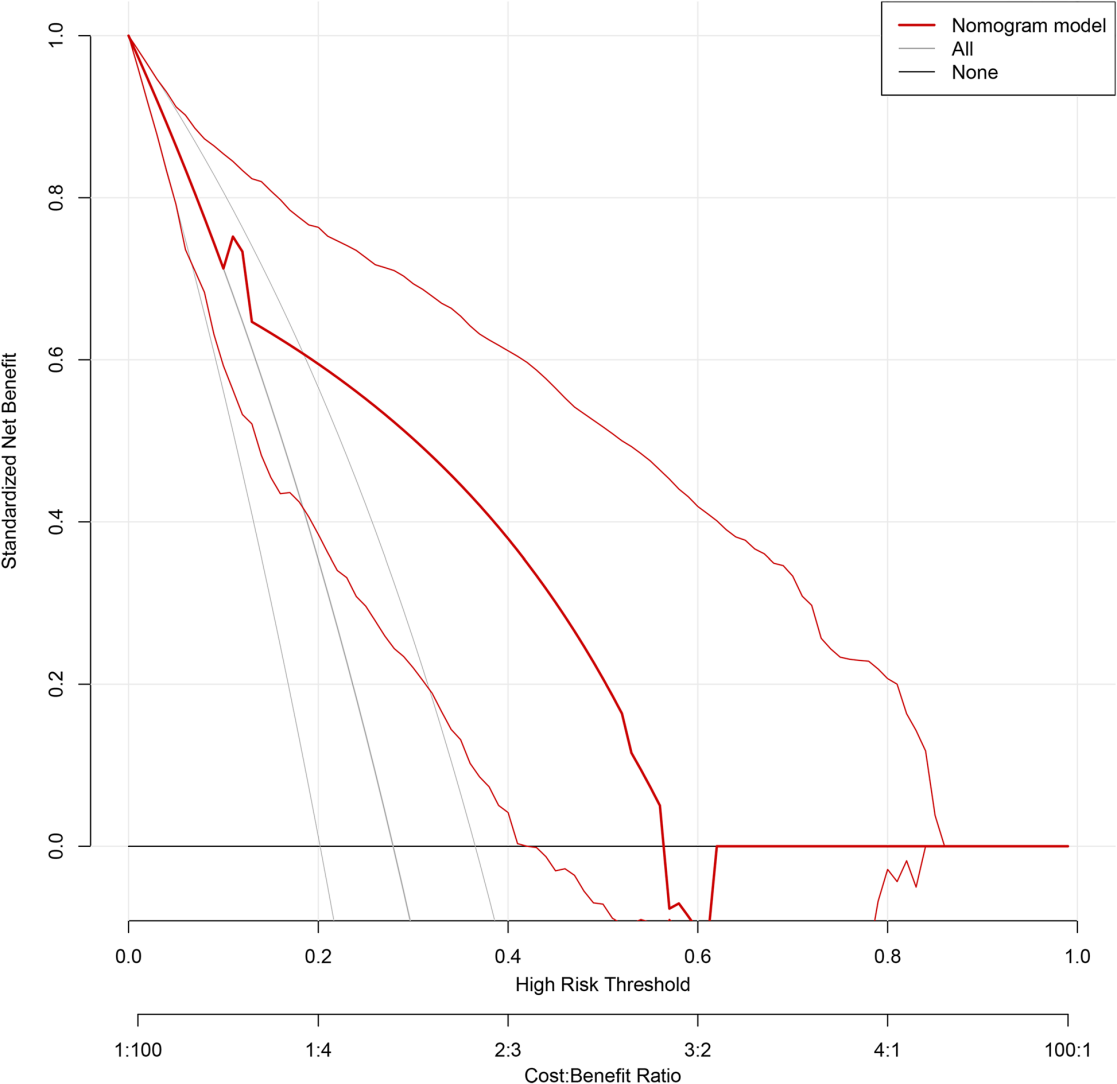
Decision curve analysis for the nomogram model in the validation set

The validation group model consisted of 104 patients. The AUC of the prediction model for the validation group was 0.783 (95% CI: 0.685–0.881) (Fig. 5 and Table 6). The C-index of the validation group was 0.783(95% CI:0.685–0.881). The AUC of model was greater than the AUCs of cortical thickness (*p* = 0.009), lymphatic gate (*p* = 0.001) and T stage (*p* = 0.007) (Table 6). The *P*-value of the Hosmer–Lemeshow-Goodness-of-Fit test was 0.783. The DCA had good net benefits in the validation group (Fig. 6).

**Table 6:** The ROC analysis of cortical thickness, hilum, T stage and the model in the validation cohort

Variable	AUC	ACC(%)	SN (%)	SP (%)	PPV (%)	NPV (%)	*P*-value	95%CI	
								**Lower**	**Upper**
Cortical_thickness	0.718	74.0	66.7	77.0	54.1	85.1	0.009	0.605	0.832
Hilum	0.639	74.0	40.0	87.8	57.1	78.3	0.001	0.514	0.764
T stage	0.614	57.6	70.0	70.0	37.5	81.3	0.007	0.496	0.731
Model	0.783	77.9	70.0	71.6	61.2	84.9		0.685	0.881

*AUC* Area under curve, *ACC* Accuracy, *SN* Sensitivity, *SP* Specificity, *PPV* Positive Predictive Value, *NPV* Negative Predictive Value, *P*-value: DeLong test of AUC, *CI* Confidence interval

## Discussion

Although US examination is currently one of the most widely used and important imaging technologies, it is not completely accurate in predicting high-burden lymph nodes [32, 33]. Predicting high lymph node burden can guide individualised treatment strategies with respect to the application of neoadjuvant chemotherapy and selection of the type of axillary surgery (SLNB *vs*. ALND) [34]. Therefore, prediction of lymph node metastasis and identification of patients with high axillary lymph node loads are both essential and challenging processes. The main strength of this study is that we successfully integrated US and clinicopathological features of lymph nodes and primary lesion ultrasound and establish a nomogram that could predict a high axillary lymph node burden.

In our study, the cortical thickness and lymphatic hilum of lymph nodes and the T stage of the primary lesion were found to be independent predictors of high-burden lymph nodes. Based on these three parameters, we established a nomogram to predict high-burden node (HBN), and our results showed that its AUC was 0.749, i.e. a satisfactory predictive value. In the nomogram, cortical thickness was more important. The point of cortical thickness, which was ≥ 3 mm, was 100 points, and thus greater than the lymphatic hilum of lymph nodes and T stage. The US characteristics of the lesion proved impossible to identify high-burden lymph nodes. However, according to the studies performed by Torstenson and Ansari, the distance between the tumour and nipple and the distance between the tumour and skin were significantly correlated with positive lymph node metastasis [35, 36]. In Yi’s study, the distance from the nipple was interconnected with high-burden lymph nodes [24]. Since this was a retrospective study, the US report failed to count the distance from the primary lesion to the nipple, which may have caused a degree of impact on the diagnostic efficiency. To our knowledge, only a limited number of studies have combined ultrasound of axillary lymph nodes, ultrasound of primary lesions and clinicopathological characteristics. Our study could comprehensively evaluate the relationship between these three parameters and high burden lymph nodes metastasis.

There are still some limitations in our research. First, this study was a retrospective study. In the axillary ultrasound report, we mainly focused on lymph nodes with large size or potential malignancies, while some lymph nodes with small size but abnormal morphology were ignored. Consequently, there may be sample selection deviation. Second, due to the small sample size, the low-burden group included a significantly greater number of cases compared to the high-burden group, which may influenced our results. Third, we did not statistically analyse the blood flow in the lymph nodes and the primary lesion. In addition, the clinical and pathological factors used in developing the nomogram were obtained postoperatively, making it challenging to directly predict the burden of lymph nodes before surgery. However, the study added information about ultrasound results, and future research may require additional studies incorporating MRI, molybdenum target and clinically assessable pathological factors before surgery and establish a preoperative prediction model. Therefore, future studies with a larger sample size and more comprehensive characteristics are expected to confirm the clinical application of the present model.

## Conclusion

In conclusion, we established a nomogram integrating the US and clinicopathological features of axillary lymph nodes and primary breast lesions to predict high-burden lymph nodes metastasis. Lymph node cortical thickness, lymphatic hilum and T stage were found to be important indicators for predicting high-burden lymph nodes metastasis, and these parameters are expected to be helpful in clinical practice in terms of reducing unnecessary ALND and identifying the patients requiring neoadjuvant chemotherapy.

## Data Availability

All data generated or analyzed during this study are included in this published article.

## Abbreviations

US: Ultrasound
ALN: Axillary lymph node
ALND: Axillary lymph node dissection
ER: Estrogen receptor
PR: Progesterone receptor
Her-2: Human epidermal growth factor receptor 2
AUC: Area under curve
ACC: Accuracy
SN: Sensitivity
SP: Specificity
PPV: Positive predictive value
NPV: Negative predictive value
CI: Confidence interval
DCA: Decision curve analysis
